# Molecular Diversity and Distribution of Whiteflies (*Bemisia tabaci*) in Cassava Fields Across South West and North Central, Nigeria

**DOI:** 10.3390/insects15110906

**Published:** 2024-11-20

**Authors:** Oghenevwairhe P. Efekemo, Olabode A. Onile-ere, Isaac O. Abegunde, Folashade T. Otitolaye, Justin S. Pita, Titus Alicai, Angela O. Eni

**Affiliations:** 1 Department of Biological Sciences, Covenant University, Ota 112233, Ogun State, Nigeria; 2 Central and West African Virus Epidemiology (WAVE) for Food Security, Covenant University Hub, Ota 112233, Ogun State, Nigeria; 3 Regional Center of Excellence for Transboundary Plant Pathogens, Central and West African Virus Epidemiology (WAVE), Pôle Scientifique et d’Innovation, Université Félix Houphouët-Boigny, Abidjan BPV 34, Côte d’Ivoire; 4 National Agricultural Research Organization (NARO), National Crops Resources Research Institute, Kampala P.O. Box 7084, Uganda

**Keywords:** *Bemisia tabaci*, whitefly, *mtCOI*, genetic diversity, haplotype, cassava mosaic disease

## Abstract

Whitefly *Bemisia tabaci* (Gennadium, Hemiptera) causes severe damage to cassava plants through excessive feeding on leaves and transmitting viruses, such as *African cassava mosaic virus* (ACMV), *East African cassava mosaic virus* (EACMV), and ipomoviruses that cause cassava brown streak disease. Currently, little is known about the molecular diversity and distribution of whitefly species in the major cassava-growing zones of Nigeria. This study aimed to address the knowledge gap by assessing the genetic diversity, distribution, and associated cassava mosaic begomoviruses (CMBs) in whiteflies across South West and North Central, Nigeria. Whitefly samples were systematically collected from cassava plants during georeferenced epidemiological surveys in 2017, 2020, and 2022. The samples were genotyped using the mitochondrial cytochrome oxidase I (*mtCOI*) marker, and CMBs were detected by PCR with virus-specific primers. Phylogenetic analyses revealed four distinct genetic groups of *B. tabaci*: Sub-Saharan Africa 1 (SSA1; 84.8%), SSA2 (1.4%), SSA3 (13.1%), and Mediterranean (MED) (0.7%). The SSA1 group was the predominant and most widely distributed genotype across the surveyed zones, with three subgroups identified: SSA1-SG1, SSA1-SG3, and SSA1-SG5. The second most frequently identified genotype, SSA3, was restricted to the North Central zone, along with the SSA2 group, which was only identified in two North Central states (Niger and Plateau). *African cassava mosaic virus* (ACMV) was detected in SSA1-SG1, SSA1-SG5, and SSA3, whereas EACMV was found in only the SSA1-SG3. The findings of this study will aid in developing better whitefly management strategies to reduce the impact of CMD on cassava production in Nigeria.

## Simple Summary

The whitefly pest, *Bemisia tabaci*, is an economically important pest given its role in the transmission of plant virus diseases such as cassava mosaic disease (CMD), which continues to devastate cassava productivity in Nigeria. Previous studies have shown that the ability of whiteflies to transmit plant viruses is dependent on several factors, chief of which is its biotype. Given that whiteflies are often morphologically indistinguishable, it is therefore important to explore the genetic diversity of whiteflies towards developing adequate management strategies for CMD in Nigeria. This study therefore investigated the genetic diversity and distribution of *B. tabaci* genotypes present in cassava farms surveyed across South West and North Central, Nigeria, while also assessing the presence of cassava mosaic begomoviruses (CMBs). The obtained whitefly samples were genotyped using the mitochondrial cytochrome oxidase I (*mtCOI*) marker. This study identified four genetic groups of *B. tabaci*, with the Sub-Saharan Africa 1 (SSA1) group being the most predominant. This research also detected African cassava mosaic virus (ACMV) and East African cassava mosaic virus (EACMV) in specific subgroups, providing insights for developing improved management strategies to mitigate cassava mosaic disease (CMD) in Nigeria.

## 1. Introduction

Cassava (*Manihot esculenta* Crantz) is an important crop for food security, given its resilience to climate change, low requirement of production inputs, and high-calorie content [1]. This staple is predominantly grown by smallholder farmers and is a critical source of sustenance for over 800 million people globally [2]. Globally, Nigeria is the largest producer of cassava [3]. However, despite a marked increase in cultivated areas over the years, the cassava yield in Nigeria remains low (8.2 tons/hectare in 2019) [3]. The cassava yield is significantly impacted by pests and diseases, with cassava mosaic disease (CMD) being the most significant disease, causing an estimated 30–40% yield reduction in Nigeria [4,5,6]. CMD is caused by a group of eleven *cassava mosaic begomoviruses* (CMBs), with only two of them, the *African cassava mosaic virus* (ACMV) and the *East African cassava mosaic virus* (EACMV), occurring in Nigeria [7]. These viruses are transmitted through infected cassava planting materials (stem cuttings) and/or by the whitefly vector, *B. tabaci* (Genaddius), which is also a destructive pest of cassava [7,8]. Whiteflies damage cassava plants through direct phloem feeding and through the excretion of honeydew, which facilitates the growth of saprophytic fungi, such as *Capnodium* spp. These fungi appear as a black sooty mold on cassava leaves, limiting access to sunlight and thus reducing photosynthesis and tuber yield [9].

In Africa, high populations of *B. tabaci* have been linked to outbreaks of various plant virus diseases [9]. For example, in Uganda, a significant increase in whitefly numbers (>200 whiteflies on the top five leaves of cassava plants) was identified as a key factor in the severe CMD pandemic of the 1990s. Similarly, high whitefly populations were recorded during the 2004 outbreaks of cassava brown streak disease (CBSD) in south-central Uganda, western Kenya, and northwestern Tanzania [10]. The reasons for the sudden increase in whitefly population during the CMD and CBSD pandemic are yet to be understood. However, the continuous monitoring of the whitefly population in cassava-growing areas is critical to the management of the virus diseases they spread.

The *B. tabaci* complex encompasses over 40 morphologically indistinguishable species; hence, an effective management of viral disease spread would require an accurate understanding of the *B. tabaci* species involved [11]. Molecular markers, such as the mitochondrial cytochrome oxidase I (*mtCOI*) gene, provide a reliable means for differentiating *B. tabaci* species [12]. In Africa, 19 *B. tabaci* genotype groups have been identified, namely the Sub-Saharan Africa (SSA) groups 1 to 13 (SSA1 to SSA13), Uganda, New-World 1, MED, MEAM1, Indian Ocean, and Italy1 [13]. The SSA groups are the primary cassava colonizers, each with its own unique distribution and subgroups [14,15]. In Nigeria, extensive whitefly genetic diversity studies have been conducted in the South East and South South zones [16], while, in the South West, recent studies by Akintola et al. [17] only covered four of the six states (Oyo, Osun, Ogun, and Kwara). There is still limited information about the distribution of whitefly species in other zones of Nigeria, including the major cassava-producing and planting material distribution Benue state. The main objective of this study was to assess the genetic diversity and distribution of the *B. tabaci* complex across states in the South West zone (Ekiti, Lagos, Ogun, Ondo, Osun, and Oyo states) and the North Central zone (Benue, Kogi, Kwara, Nassarawa, Niger, and Plateau states) of Nigeria.

## 2. Materials and Methods

### 2.1. Study Area, Field Selection, and Sampling Criteria

This study was conducted in Nigeria, which is the highest cassava-producing country in the world. Nigeria comprises 36 states and a federal capital territory (Abuja). The states are grouped into six geopolitical zones for administrative and political purposes (North Central, North East, North West, South East, South West, and South South). Cassava is cultivated in 24 out of the 36 states in Nigeria, 50% of which are situated in the North Central and South West zones [18].

Cassava epidemiological field surveys were conducted in 2017, 2020, and 2022 across 12 states in 2 geo-political zones of Nigeria: the South West zone (Ekiti, Lagos, Ogun, Ondo, Osun, and Oyo states) and the North Central zone (Benue, Kogi, Kwara, Nassarawa, Niger, and Plateau states). Surveyed fields in the South West Zone were located between latitudes 6° and 8° N and longitudes 2° and 5° E, while the North-Central Zone lay between latitudes 6° and 10° N and longitudes 4° and 9° E. The majority of the surveyed states (Benue, Ekiti, Kwara, Kogi, Nassarawa, Ogun, Ondo, Osun, Oyo, and Plateau states) are located in the derived savanna agroecological zone of Nigeria. Few surveyed states are situated in the humid forest zone (Lagos, Ondo, and Osun) and the southern Guinea savanna zone (Niger).

Fields for sample collection were selected at 10–20 km intervals depending on the availability of cassava farms along main and rural roads, as described by [19]. Adult whiteflies were collected from the top five leaves of several cassava plants in each sampled field using an aspirator and stored in a microcentrifuge tube (Thermo Fisher Scientific, Waltham, MA, USA) containing molecular grade (99.5%) ethanol. The geo-coordinates per field were obtained using a geographical positioning system (Garmin, Olathe, KS, USA).

### 2.2. Whitefly DNA Extraction

DNA was extracted from a single whitefly randomly selected from each sample tube. Using the standard Chelex extraction method described by Mugerwa et al. [9], the whitefly was placed in a 1.5 mL microcentrifuge tube containing 50 µL of 10% (*w*/*v*) Chelex 100 resin solution. The whitefly was crushed using a sterile plastic rod to extract genomic DNA. The mixture was incubated at 56 °C for 20 min, then 100 °C for 5 min before centrifugation at 13,500× *g* for 5 min. The supernatant was collected using a micropipette, transferred to a sterile microcentrifuge tube, and stored at −20 °C until further use.

### 2.3. Mitochondrial COI (mtCOI) Amplification and Sequencing

For the genotyping analysis, the mitochondrial cytochrome C oxidase I (*mtCOI*) gene of each whitefly was amplified using the primer pair 2195Bt (5′-TGRTTTTTTGGTCATCCRGAAGT-3′) and C012/Bt-sh2 (5′-TTTACTGCACTTTCTGCC-3′) [9]. The PCR reaction mixture (50 µL) contained 10× PCR reaction buffer, 10 mM dNTPs, 10 µM of each primer, and 1 U Taq DNA Polymerase. The reaction cycle for the 2195Bt and C012/Bt-sh2 primer pair was an initial denaturation of 94 °C for 2 min, 35 cycles of denaturation at 94 °C for 20 s, annealing at 52 °C for 30 s, elongation at 72 °C for 1 min, final extension of 10 min at 72 °C, and hold at 4 °C. The PCR products were resolved on 1% (*w*/*v*) agarose gel in 1× TAE stained with ethidium bromide (10 mg/mL) and visualized using a UV gel documentation system (UV light 302 nm) (Analytik Jena, Jena, Germany). Amplicons with the expected band size were purified using the Easytaq DNA purification kit (TransGen Biotech, Beijing, China) following the manufacturer’s instructions and sequenced using the Sanger method (Inqaba Biotech, Ibadan, Nigeria).

### 2.4. Determination of Whitefly Genotype and Phylogenetic Analyses

Obtained sequences that included the *mtCO1* 3′ barcoding region [20] were quality checked and trimmed, and low-quality sequences or sequences having too many indels were removed before future analyses. An available whitefly genotype database from NCBI GenBank was curated and stored locally, after which high quality sequences obtained in this study were compared against the stored database using the standalone BLAST version 2.13.0+ for Windows. A neighbor-joining tree with 1000 replications was computed to visualize the relationships between the whitefly samples obtained in this study and those obtained from the GenBank using Geneious Prime 2024.0 [21]. A 3.5% divergence limit was used as the threshold for species delimitation [22], while, within SSA1 species, subgroups were designated based on >0.6% divergence in partial *mtCO1* sequences as reported by Mugerwa et al. [23] and Legg et al. [24]. Distribution maps were generated from the collected field GPS data using sf package in R version 4.2.2.

### 2.5. Population Genetic Analysis

The extent of genetic differences amongst nucleotide sequences of the *B. tabaci* isolates identified in this study was examined. Estimates were obtained for the number of haplotypes, polymorphic sites (S), average number of nucleotide differences (k), nucleotide diversity (Pi), and haplotype diversity (Hd) using the mismatch distribution procedure of DnaSP Ver. 5.10.01 [25]. Tajima’s D and Fu’s Fs were also obtained using DnaSP Ver. 5.10.01 to determine whether the sampled whitefly populations were stable or expanding.

### 2.6. Polymerase Chain Reaction (PCR) Amplification for CMB Detection

The DNA extracted from each whitefly sample was also screened for the presence of CMBs using specific CMB detection primers (Table S1). PCR assay was carried out using the protocol as described by Matic et al. [26]. PCR reaction composition was as follows: PCR reaction buffer (10×), 2.5 mM dNTPs, 10 μM of each primer, 5 U Taq DNA polymerase (TransGen Biotech, Beijing, China), diluted DNA, and sterile distilled water to a final volume. The general reaction cycles were an initial denaturation at 94 °C for 2 min followed by 30 cycles of denaturation at 94 °C for 1 min, annealing at 55 °C for 1 min, extension at 72 °C for 1 min, and a final extension of 72 °C for 10 min. The amplified PCR products were resolved on a 1% agarose gel stained with ethidium bromide (10 mg/mL) alongside a 1 kbp plus DNA ladder (Thermo Fisher Scientific, MA, USA) using a gel electrophoresis system (BioRad, CA, USA) at 100 V for about 40 min. The gel was visualized using a UV gel documentation system (UV light 302 nm) (Analytik Jena, Jena, Germany).

## 3. Results

### 3.1. Genetic Diversity of Whiteflies

**Figure 1:**
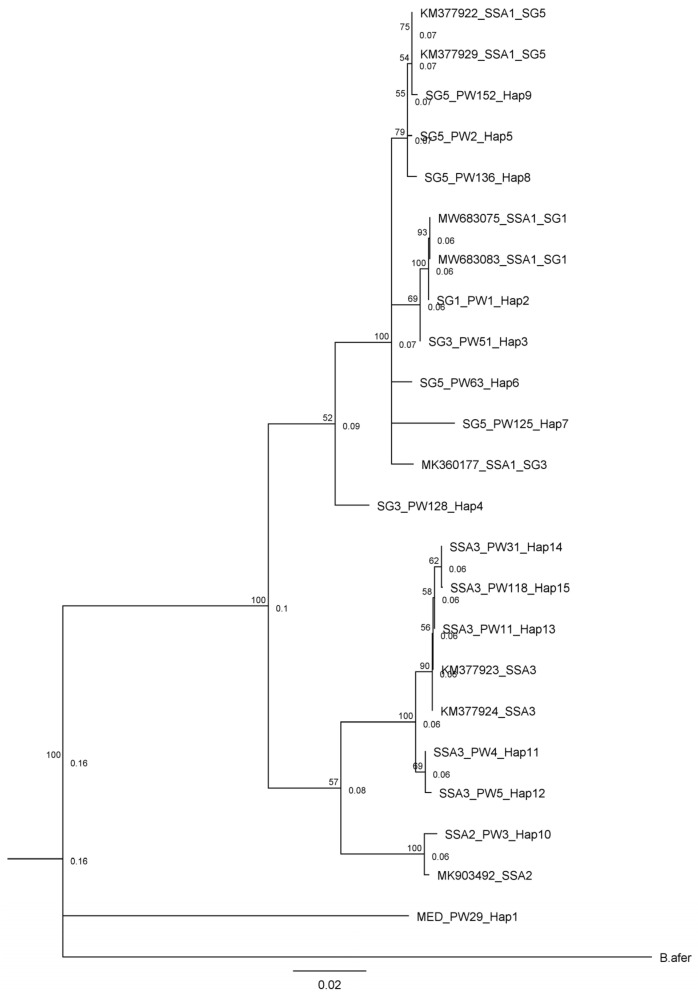
Phylogenetic tree of haplotypes obtained in this study alongside representative whitefly biotypes from the NCBI GenBank and *Bemsia afer* as outgroup (a list of sequences belonging to each haplotype can be found in Supplementary Materials).

Whiteflies were collected from a total of 196 fields across South West and North Central in 2017, 2020, and 2022. The number and distribution of the whitefly collection sites varied based on the presence and abundance of whiteflies in cassava farms across the surveyed zone in various years. Following the cleanup of the sequence data, a total of 145 high-quality *mtCO1* gene sequences were obtained. The identified sequences were submitted to the GenBank database in NCBI, with accession numbers from OR807570 to OR807714. Phylogenetic analyses of the obtained sequences revealed that whiteflies collected during this study belonged to four *B. tabaci* genotype groups (namely SSA1 (84.8%), SSA2 (1.4%), SSA3 (13.1%), and MED-ASL (0.7%) groups) (Figure 1). Of the three SSA1 genotype subgroups identified, SSA1-SG5 was the most abundant, comprising 82.1% of the SSA1 whiteflies identified in this study, followed by SSA1-SG1 (16.3%) and SSA1-SG3 (1.6%).

The whitefly sequences from this study had between 98.1 and 100% nucleotide (nt) similarity to previously published sequences in the GenBank (Table 1). The highest pairwise similarity was found among members of the SSA1-SG5, where 92 of the 101 members of this group identified in this study had 100% nucleotide identity with GenBank sequences from Oyo State, South West, Nigeria. Additionally, the isolates SSA1-SG1, SSA1-SG3, SSA2, and MED-ASL in this study clustered with isolates from Uganda with 98% to 100% nucleotide identity. The SSA3 genotype identified was most closely related (99.3–100% nucleotide identity) to isolates from Eastern Nigeria. The genetic variation of the *mtCO1* gene sequences obtained in this study ranged from 0.0% to 9.7% (Table 2) and the isolates showed both intraspecific and interspecific variations. The intraspecific variations for SSA1-SG1, SSA1-SG5, and SSA3 were 0.6%, 0.3%, and 0.5%, respectively. Intraspecific variations for SSA1-SG3, SSA2, and MED-ASL were not computed because of the few numbers of these groups identified in the current study. The interspecific variation between SSA1 and SSA2 was 7.8%; between SSA1 and SSA3 was 6.9%; and the lowest interspecific variation (5.7%) was between SSA2 and SSA3.

### 3.2. Population Genetic Diversity

Population genetics analysis of *B. tabaci* genetic groups revealed the presence of 15 unique *mtCO1* haplotypes (Table 3). In total, 96 polymorphic sites (S) were identified and the haplotype diversity (h) of the entire sample population of 145 whitefly sequences from South West and North Central, Nigeria was 0.530 and the nucleotide diversity (π) presented values of 0.02381 (Table 3). Tests for neutrality on the entire population (Tajima’s D = −1.46119 with *p* > 0.10) suggest a recent population expansion. However, since the *p*-value is greater than 0.10, this deviation is not statistically significant.

### 3.3. Distribution of Identified Whitefly Genotypes Across the Surveyed Zones

**Figure 2:**
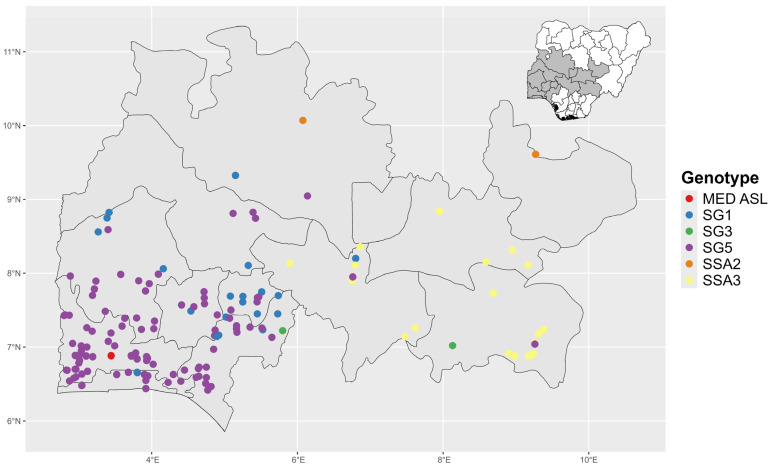
Distribution of whitefly (*B. tabaci*) genotypes in cassava farms in South West and North Central, Nigeria. SSA—Sub-Saharan African. SG1—SSA1 subgroups 1; SG3—SSA1 subgroups 3; SG5—SSA1 subgroups 5.

Among the Sub-Saharan African (SSA) genotype groups, the SSA1 genotype group was the most widespread as it was found in cassava fields in both South West and North Central states (Figure 2). SSA1-SG5 was observed in 10 out of 12 states in South West and North Central, while SSA1-SG1 was observed in 7 out of the 12 states surveyed (Table 4). In contrast, SSA2 and SSA3 genotypes were restricted to cassava fields in the North Central zone of Nigeria. SSA2 was observed in two states in the North Central zone (Niger and Plateau) and SSA3 was found in three states (Benue, Kogi, and Nasarawa). The MED-ASL was the least prevalent *B. tabaci* genotype identified and was only found in one location in the South West zone (Ogun state).

### 3.4. Viruses Detected in Whiteflies Sampled from Cassava in South West and North Central, Nigeria

ACMV was detected in 12.4% of the whitefly samples identified in this study, while EACMV was detected in only 0.7% (Table 5). ACMV was detected in three of the whitefly genotype groups (SSA1-SG1, SSA1-SG5, and SSA3) identified in this study, whereas EACMV was found in only the SSA1-SG5 group (Table 5).

## 4. Discussion

This study presents the molecular diversity and distribution of whiteflies (*B. tabaci*) colonizing cassava plants across South West and North Central, Nigeria. The whitefly isolates were genotyped using the *mtCO1* molecular maker. Four *B. tabaci* genetic groups—SSA1, SSA2, SSA3, and MED—were identified as being present within the surveyed area in Nigeria. Population genetic analysis in this study indicated a moderate genetic diversity within the population and a test of neutrality indicated a population expansion; however, further evidence is needed to confirm this hypothesis.

Among the *B. tabaci* whitefly genotype species identified, SSA1 was the most widespread, occurring in 10 out of the 12 states sampled, indicating that this genotype has a wide adaptation to diverse vegetation and agroecological types. Previous studies conducted in East Africa showed that the high prevalence of the SSA1 whitefly genotype is often linked to its wide host range, which includes food crops such as common bean, cowpea, pumpkin, tomato, and eggplant [27]. In addition, weeds such as *Erythrina abyssinica* and *Sida acuta* have been cited as alternate hosts of the SSA1 whitefly genotype [9] (Mugerwa et al., 2021). Smallholder farmers often practice intercropping in a bid to improve soil fertility, maximize the land, and as a cultural practice for the management of pests and diseases [28]. This practice may explain the high prevalence of SSA1 genotypes given the availability of suitable host plants.

We also identified three SSA1 subgroups: SSA1-SG1, SSA1-SG3, and SSA1-SG5. Previous studies conducted in Nigeria by Akintola et al. [17], Nwezeobi et al. [16], and Ghosh et al. [29] reported that SSA1-SG1 and SSA1-SG5 genotypes were the predominant whitefly genotypes in Nigeria, similar to the findings from this study. Notably, SSA1-SG1 is the most prevalent SSA1 subgroup in many cassava-growing regions in East and Central Africa, including the Democratic Republic of the Congo (DRC) and neighboring countries such as Burundi, the Central African Republic, Rwanda, Tanzania, and Uganda [10]. In addition, SSA1-SG1 has been associated with high whitefly abundance and severe CMD and CBSD pandemics [5,30,31]. However, in West African countries, including Nigeria where SSA1-SG1 has been reported, there have been no incidences of high whitefly abundance (>200 whiteflies counted on the top five leaves of the cassava plant) [7,16]. Given the threat of the westward spread of CBSD from East and Central Africa, the continued monitoring of whitefly abundance in Nigeria is vital for rapid response in the event of a CBSD outbreak and the biology of the whitefly genotype.

The second most dominant whitefly genotype identified in this study (SSA3) was restricted to cassava farms in North Central, Nigeria. A previous study by Nwezeobi et al. [16] observed that, although SSA3 occurred in relatively low numbers, SSA3 was the most widespread and prevalent genotype in eastern Nigeria. In contrast with both this study and the work by Nwezeobi et al. [16], a previous study in Nigeria by Akintola et al. [17] did not detect the SSA3 genotype group. The reason for the clustering pattern of SSA3 around North Central and the eastern part of Nigeria seems to be unclear; however, it could be attributed to factors such as the types of varieties of cassava planted and/or other agroecological characteristics. This can be a useful aspect of consideration for further studies. Notably, SSA3 has been reported in other West and Central African countries, including Benin, Togo, Cameroon, Central African Republic, and the Democratic Republic of the Congo, but it is rarely reported in East Africa [32,33]. Namuddu et al. [33] reported the first instance of SSA3 in Uganda and suggested that SSA3 might have been introduced from West Africa. Very little is known about SSA3 regarding its virus transmission ability, mating combability with other whitefly genotypes, fecundity, and response to insecticides; however, it has been observed in cassava fields with cassava mosaic disease (CMD) [16].

The SSA2 genotype group was the least prevalent SSA group identified in this study and was the only genotype group identified in Plateau State. Previous studies in Nigeria [16,17,29] have not reported the SSA2 genotype group. The low occurrence of SSA2 in Nigeria is contrary to what has been reported in other cassava-cultivating countries in East, Southern, and Central Africa [14,15,27,34]. SSA2 was first recorded as the predominant whitefly during the CMD pandemic in 1997; however, in subsequent years, the frequencies of SSA2 significantly reduced from 63.9% to 1.4% [24]. SSA2 was identified at only one location in this study; hence, we cannot state whether the occurrence of SSA2 is an isolated event due to the movement of whitefly nymphs. Further studies are therefore required to explore the spread and distribution of SSA2 in North Central, Nigeria.

In addition to the Sub-Saharan (SSA) genotype groups (SSA1, SSA2, and SSA3), our study also identified the presence of the Mediterranean (MED) genotype group. The MED genotype group has been recognized as a highly invasive species that has extended its presence throughout Africa and other continents [13,35]. Invasive *B. tabaci* species such as MED have been reported to exhibit greater efficiency in virus transmission, adaptability to temperature changes, and resistance to insecticides when compared with non-invasive species [36]. In addition, the MED genotype group is referred to as a highly polyphagous genotype of the *B. tabaci* complex, infesting over 600 different plant species. However, studies have indicated that cassava is not a preferred host plant for MED whiteflies [37]. Sartor et al. [37] reported that MED whiteflies were unable to lay viable eggs and survive on cassava plants for longer than 2 days. Thus, the low proportion of MED identified in this study could be because whiteflies were collected strictly from cassava plants. Similar to this study, Akintola et al. [17] and Nwezeobi et al. [16] also reported low proportions of MED in their respective studies.

The *B. tabaci* complex is of economic importance because of its role as a vector of both cassava mosaic begomovirus (CMB) and cassava brown streak virus (CBSV) [8,13]. Both ACMV and EACMV were detected in the whitefly isolates collected in this study. In previous studies in Nigeria, neither Akintola et al. [17] nor Nwezeobi et al. [16] detected any CMBs in the whitefly specimens collected in their studies. In this study, ACMV was detected in SSA1-SG1, SSA1-SG5, and SSA3, while EACMV was detected in SSA1-SG5. Although virus detection in the *B. tabaci* samples does not necessarily confirm its ability to transmit the virus to host plants, the presence of the virus in the whitefly implies that the whitefly can acquire the virus and has the potential to spread the virus to host plants.

## 5. Conclusions

This study provides valuable insights into the molecular diversity of whitefly species colonizing cassava plants and their distribution across South West and North Central, Nigeria. The results reveal the presence of four *B. tabaci* species, namely SSA1, SSA2, SSA3, and MED. Findings also show the predominance of SSA1 across the surveyed zones, the unique distribution of SSA3 in the North Central zone, and the low proportion of SSA2 and MED. In addition, the presence of CMBs in the SSA1 and SSA3 whitefly genotype emphasizes their role as vectors of cassava mosaic disease. These findings would be useful for both researchers and breeders for the development of whitefly management strategies for the study location.

## Figures and Tables

**Table 1:** Nucleotide similarities among whitefly (*B. tabaci*) isolates obtained using the primer pair 2195Bt and C012/Bt-sh2 to sequences obtained from NCBI GenBank.

Whitefly Genotype		Number of Isolates	Nucleotide Identity Similarity %	Accession Numbers	Country
SSA1	SG1	20	99.87–100	MW683075 MW683083	Uganda
	SG3	2	98.55–99.09	MK360177	Uganda
	SG5	101	98.15–100	KM377922 KM377929	Nigeria
	SSA1 Subtotal	123			
SSA2		2	99.45–99.64	MK903492	Uganda
SSA3		19	99.34–100	KM377923 KM377924 KM377926	Nigeria
MED		1	100	MH205754	Uganda

**Table 2:** Genetic variation of whitefly (*B. tabaci*) mitochondrial cytochrome oxidase I (*mtCO1*) gene sequences obtained using the primer pair 2195Bt and C012/Bt-sh2.

Whitefly Genotype	SSA1-SG1 (%)	SSA1-SG5 (%)	SSA2 (%)	SSA3 (%)
SSA1-SG1 (Range)	0.15 (0.00–0.52)			
SSA1-SG5 (Range)	1.66 (1.28–3.79)	0.33 (0.00–3.27)		
SSA2 (Range)	7.20 (7.15–7.37)	7.88 (7.24–9.67)	a	
SSA3 (Range)	6.71 (6.51–7.24)	6.97 (6.28–8.5)	5.67 (5.49–6.00)	0.53 (0.00–0.99)

a = intraspecific genetic variation for SSA2 genotype was not computed because there were only two SSA2 isolates in this study.

**Table 3:** Population genetic analysis of whitefly (*B. tabaci)* genotypes colonizing cassava in South West and North Central, Nigeria.

Whitefly Genotype	*n*	h	S	K	Hd	π	Fu’s Fs	Tajima’s D	*p* Value
SG1	20	4	5	0.589	0.363	0.00078	−1.197	−1.78003	0.10 > *p* > 0.05
SG3	2	2	31	31.000	1.000	0.03985	3.434	-	-
SG5	101	5	4	0.079	0.078	0.00018	−7.421	−1.77619	0.10 > *p* > 0.05
SSA1 subtotal	123	8	24	2.288	0.354	0.00512	1.354	−1.47570	*p* > 0.10
SSA2	2	2	2	2.000	1.000	0.00261	0.693	-	-
SSA3	19	5	7	2.503	0.6491	0.00412	1.243	0.83356	*p* > 0.10
MED	1	1	-	-	-	-	-	-	-
Total	145	15	96	10.645	0.530	0.02381	8.918	−1.46119	-

*n*—number of sequences; h—number of haplotypes; S—number of polymorphic sites; K—average number of nucleotide differences; Hd—haplotypic diversity; π—nucleotide diversity; Fu’s Fs and Tajima’s D—neutrality test.

**Table 4:** Distribution of whitefly genotypes across surveyed zones in South West and North Central, Nigeria.

Zone	State (*n*)	SSA1-SG1 %	SSA1-SG3 %	SSA1-SG5 %	SSA2 %	SSA3 %	MED-ASL %
North Central	Benue (12)	0.00	8.33	8.33	0.00	83.33	0.00
	Kogi (8)	12.50	0.00	12.50	0.00	75.00	0.00
	Kwara (4)	25.00	0.00	75.00	0.00	0.00	0.00
	Nasarawa (3)	0.00	0.00	0.00	0.00	100.00	0.00
	Niger (3)	33.33	0.00	33.33	33.33	0.00	0.00
	Plateau (1)	0.00	0.00	0.00	100.00	0.00	0.00
	Subtotal (31)	9.68	3.23	19.35	6.45	61.29	0.00
South West	Ekiti (9)	55.56	0.00	44.44	0.00	0.00	0.00
	Lagos (10)	10.00	0.00	90.00	0.00	0.00	0.00
	Ogun (40)	0.00	0.00	97.50	0.00	0.00	2.50
	Ondo (26)	23.08	3.85	73.08	0.00	0.00	0.00
	Osun (8)	12.50	0.00	87.50	0.00	0.00	0.00
	Oyo (21)	19.05	0.00	80.95	0.00	0.00	0.00
	Subtotal (114)	14.91	0.88	83.33	0.00	0.00	0.88
Grand Total (145)		13.79	1.38	69.66	1.38	13.10	0.69

**Table 5:** Proportion of CMBs identified in different whitefly genotypes collected from cassava farms in South West and North Central, Nigeria.

Whitefly Genotype	ACMV	EACMV
SSA1-SG1	15% (3/20)	0
SSA1-SG3	0	0
SSA1-SG5	10.9% (11/101)	1% (1/101)
SSA2	0	0
SSA3	21.1% (4/20)	0
MED	0	0
Total	12.4% (18/145)	0.7% (1/145)

## Data Availability

Partial *mtCO1* sequences of the *B. tabaci* whiteflies obtained in this study were deposited in GenBank under accession numbers OR807570 to OR807714.

## Supplementary Materials

The following supporting information can be downloaded at: https://www.mdpi.com/article/10.3390/insects15110906/s1, Table S1: Details of the primer pairs used for the detection of *Cassava mosaic begomoviruses* (CMBs) [26,38,39].
