# Supplementary material for: Molecular Diversity and Distribution of Whiteflies (Bemisia tabaci) in Cassava Fields Across South West and North Central, Nigeria
Source: Insects. 2024 Nov 20;15(11):906. doi: 10.3390/insects15110906 (PMC11594662; doi:10.3390/insects15110906)
Supplement: Supplementary file 1 [file insects-15-00906-s001.zip › insects-3227905-supplementary.pdf]

Table S1. Details of the primer pairs used for the detection of *Cassava mosaic begomoviruses* (CMBs).

	Primer name	Primer sequences (5' to 3')	Target	Size	Reference
1.	JSP 001<br>JSP 002	ATGTCGAAGCGACCAGGAGAT<br>TGTTTATTAATTGCCAATACT	ACMV	783 bp	Pita et al. [1]
2.	ACMVBF<br>ACMVBR	TCGGGAGTGATACATGCGAAGGC<br>GGCTACACCAGCTACCTGAAGCT	ACMV	628 bp	Matic et al. [2]
3.	JSP 001<br>JSP 003	ATGTCGAAGCGACCAGGAGAT<br>CCTTTATTAATTTGTCACTGC	EACMV	780 bp	Pita et al. [1]
4.	CMBRepF<br>EACMVRepR	CRT CAA TGA CGT TGT ACC A<br>GGT TTG CAG AGA ACT ACA TC	EACMV	650 bp	Alabi, Kumar & Naidu [3]

1. Pita, J. S.; Fondong, V. N.; Sangaré, A.; Otim-Nape, G. W.; Ogwal, S.; Fauquet, C. M. Recombination, Pseudorecombination and Synergism of Geminiviruses Are Determinant Keys to the Epidemic of Severe Cassava Mosaic Disease in Uganda. *Journal of General Virology* **2001**, *82* (3), 655–665. https://doi.org/10.1099/0022-1317-82-3-655.
2. Matic, S.; Pais da Cunha, A. T.; Thompson, J. R.; Tepfer, M. Short Communication an Analysis of Viruses Associated with Cassava Mosaic Disease in Three Angolan Provinces. *J Plant Pathol.* **2012**, *94* (2), 443–450. https://doi.org/10.4454/JPP.FA.2012.043.
3. Alabi, O. J.; Kumar, P. L.; Naidu, R. A. Multiplex PCR for the Detection of African Cassava Mosaic Virus and East African Cassava Mosaic Cameroon Virus in Cassava. *Journal of Virological Methods* **2008**, *154* (1–2), 111–120. https://doi.org/10.1016/j.jviromet.2008.08.008.

Haplotype information

Number of haplotypes, h: 15
Haplotype diversity, Hd: 0.5300
Hap_1: 1 [1]
Hap_2: 20 [2-21]
Hap_3: 1 [22]
Hap_4: 1 [23]
Hap_5: 97 [24-62 64-88 91-94 96-124]
Hap_6: 1 [63]
Hap_7: 1 [89]
Hap_8: 1 [90]
Hap_9: 1 [95]
Hap_10: 2 [125-126]
Hap_11: 2 [127 135]
Hap_12: 11 [128-133 136 140-143]
Hap_13: 3 [134 138 145]
Hap_14: 1 [137]
Hap_15: 2 [139 144]

Hap_1: 1 [MED_PW29]
Hap_2: 20 [SG1_PW1 SG1_PW33 SG1_PW47 SG1_PW49 SG1_PW52 SG1_PW53 SG1_PW55 SG1_PW61 SG1_PW81 SG1_PW87 SG1_PW112 SG1_PW113 SG1_PW115 SG1_PW134 SG1_PW139 SG1_PW142 SG1_PW144 SG1_PW145 SG1_PW154 SG1_PW194]
Hap_3: 1 [SG3_PW51]
Hap_4: 1 [SG3_PW128]
Hap_5: 97 [SG5_PW2 SG5_PW13 SG5_PW14 SG5_PW15 SG5_PW16 SG5_PW17 SG5_PW18 SG5_PW19 SG5_PW20 SG5_PW21 SG5_PW22 SG5_PW23 SG5_PW24 SG5_PW25 SG5_PW26 SG5_PW27 SG5_PW28 SG5_PW34 SG5_PW35 SG5_PW36 SG5_PW37 SG5_PW38 SG5_PW39 SG5_PW40 SG5_PW41 SG5_PW42 SG5_PW43 SG5_PW44 SG5_PW45 SG5_PW46 SG5_PW48 SG5_PW50 SG5_PW54 SG5_PW56 SG5_PW57 SG5_PW58 SG5_PW59 SG5_PW60 SG5_PW62 SG5_PW64 SG5_PW65 SG5_PW66 SG5_PW67 SG5_PW68 SG5_PW69 SG5_PW70 SG5_PW71 SG5_PW72 SG5_PW73 SG5_PW74 SG5_PW75 SG5_PW76 SG5_PW77 SG5_PW78 SG5_PW79 SG5_PW80 SG5_PW82 SG5_PW83 SG5_PW86 SG5_PW106 SG5_PW108 SG5_PW114 SG5_PW116 SG5_PW117 SG5_PW143 SG5_PW148 SG5_PW149 SG5_PW150 SG5_PW153 SG5_PW155 SG5_PW157 SG5_PW158 SG5_PW160 SG5_PW161 SG5_PW162 SG5_PW163 SG5_PW164 SG5_PW165 SG5_PW167 SG5_PW169 SG5_PW170 SG5_PW172 SG5_PW173 SG5_PW176 SG5_PW178 SG5_PW180 SG5_PW185 SG5_PW186 SG5_PW187 SG5_PW188 SG5_PW189 SG5_PW190 SG5_PW191 SG5_PW192 SG5_PW193 SG5_PW195 SG5_PW196]
Hap_6: 1 [SG5_PW63]
Hap_7: 1 [SG5_PW125]
Hap_8: 1 [SG5_PW136]
Hap_9: 1 [SG5_PW152]
Hap_10: 2 [SSA2_PW3 SSA2_PW119]
Hap_11: 2 [SSA3_PW4 SSA3_PW12]
Hap_12: 11 [SSA3_PW5 SSA3_PW6 SSA3_PW7 SSA3_PW8 SSA3_PW9 SSA3_PW10 SSA3_PW30 SSA3_PW123 SSA3_PW126 SSA3_PW127 SSA3_PW129]
Hap_13: 3 [SSA3_PW11 SSA3_PW32 SSA3_PW135]
Hap_14: 1 [SSA3_PW31]

Hap_15: 2 [SSA3_PW118 SSA3_PW133]
